# Impact of fluoroquinolone cascade reporting of urine samples on antibiotic prescribing rates in a network of urgent care clinics

**DOI:** 10.1017/ash.2022.227

**Published:** 2022-06-20

**Authors:** Brittani M. Weichman, Amanda M. Bushman, Kathie L. Rogers, Rossana Rosa

**Affiliations:** 1 Department of Pharmacy, UnityPoint Health, Des Moines, Iowa; 2 Pathology Laboratory, Des Moines, Iowa; 3 Department of Medicine, University of Iowa-Des Moines, Des Moines, Iowa; 4 Infectious Diseases Service, UnityPoint Health, Des Moines, Iowa (Present affiliations: Microbiology Laboratory, Nebraska Medicine, Omaha, Nebraska [K.L.R.] and Department of Quality and Patient Safety, Jackson Health System, Miami, Florida [R.R.])

## Abstract

Cascade reporting is an antimicrobial stewardship strategy that has been successfully implemented in inpatient settings, but evidence of its impact on outpatient settings is scarce. We report on the impact on fluroquinolone prescribing at a network of urgent care clinics following the implementation of cascade reporting of Enterobacterales in urine cultures.

Cascade susceptibility reporting is defined by the Infectious Diseases Society of America (IDSA) as a type of selective reporting in which antimicrobial susceptibility results of secondary antibiotics are only reported if an organism is resistant to primary antibiotic choices within the antibiotic class.^1^ The use of cascade reporting has been supported by the IDSA guidelines as an element of an antimicrobial stewardship program.^1^ Currently, however, there is no standardized guidance for the implementation of cascade reporting by microbiology laboratories.

More than 50% of antibiotic expenditures in the United States occur in the outpatient setting.^2^ Furthermore, the number of urgent care clinic visits has rapidly increased in the United States^3^ and significant antibiotic prescribing variability has been noted in this setting.^4^ Urgent care clinics have seldom been the target of antimicrobial stewardship interventions.^5^ Therefore, the objective of our study was to evaluate the impact of cascade reporting of fluoroquinolone antimicrobial susceptibility on antibiotic prescribing rates in urgent care and express care clinics.

## Methods

**Fig. 1.**
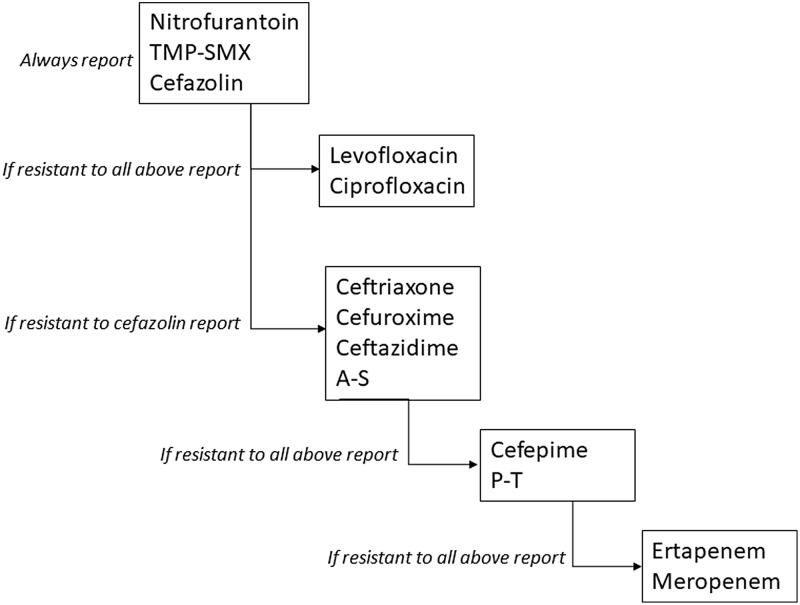
Antimicrobial susceptibility reporting rules for urine cultures with growth of Enterobacterales. Note. TMP-SMX, trimethoprim-sulfamethoxazole; A-S, ampicillin-sulbactam; P-T, piperacillin-tazobactam.

The intervention was conducted at a network of urgent care and express care clinics that are part of an integrated health system in Des Moines, Iowa. Urine samples are collected in the clinics and processed for culturing and susceptibility testing at an off-site centralized microbiology laboratory. Once available, urine culture results are displayed in the electronic medical record (Epic, Verona, WI). Prior to the intervention, cascade reporting was in place for urine cultures with growth of Enterobacterales and was limited to third- and fourth-generation cephalosporins, β-lactam–β-lactamase inhibitor combinations, and carbapenems. In July 2019, antimicrobial susceptibility reporting for Enterobacterales was modified to follow the rules displayed in Figure 1. Susceptibility results for trimethoprim-sulfamethoxazole, nitrofurantoin, and cefazolin were always displayed, and fluoroquinolones were reported only if the isolate was resistant to all above listed antibiotics. No changes were made to cascading rules for third- and fourth-generation cephalosporins, β-lactam–β-lactam inhibitor combinations, and carbapenems. During the month when the change in reporting started, educational meetings were held with clinic stakeholders, and informational pamphlets supporting the intervention were distributed via e-mail.

For the purposes of the study, the preintervention period was June 1, 2018, to June 30, 2019, and the postintervention period was August 1, 2019, to December 31, 2020. We performed an interrupted time series analysis (ITSA) by measuring monthly antibiotic prescriptions per 1,000 patient encounters (PE). All encounters regardless of final diagnosis were included. Data for the number of patient encounters were obtained from central administration and antibiotic prescribing data were abstracted from pharmacy prescribing records, including all initial and subsequent prescriptions. Antibiotic orders included in the analysis included all orders for fluoroquinolones, cephalexin, nitrofurantoin, and trimethoprim-sulfamethoxazole. Although we initially planned to include cefdinir and ceftriaxone in the analysis, these antibiotics were seldom used in the participating clinics and thus were not analyzed. All liquid antibiotic formulations were excluded. We decided a priori to evaluate a change in prescribing rates immediately after implementation of the intervention (change in level) as well as a change in the slope. Individual models were built for each antibiotic. Each antibiotic model was assessed for autocorrelation and seasonality. All statistical analysis were performed using Stata version 14.2 software (StataCorp, College Station, TX).

## Results

**Fig. 2.**
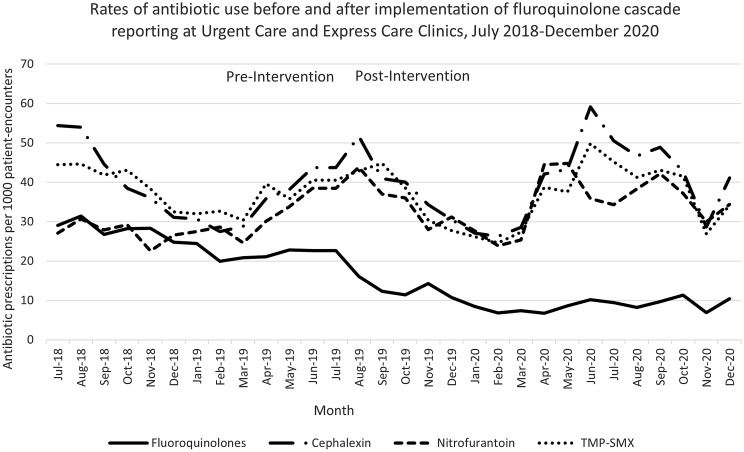
Rates of antibiotic use before and after implementation of fluoroquinolone cascade reporting at urgent care and express care clinics, July 2018–December 2020.

Data from 7 urgent care clinics and 2 express care clinics were included. Rates of antibiotic prescribing during the study period are displayed in Figure 2. In the 12 months prior to the intervention the median fluroquinolone prescribing rate was 24.6 prescriptions per 1000 PE (interquartile range [IQR], 21.89–28.86) compared to a median of 8.23 (IQR, 9.71–11.34) in the postintervention period. Estimates from the ITSA showed a 38% reduction in fluroquinolone prescribing rates following implementation of the intervention (incidence rate ratio [IRR], 95% confidence interval [CI], 0.50–0.77; *P* < .0001), and no change in slope was subsequently detected (IRR, 1.01; 95% CI, 0.98–1.04; *P* = .59).

The median prescribing rate of cephalexin was 37.10 prescriptions per 1,000 PE (IQR, 30.90–44.21) during the preintervention period. No change in cephalexin prescribing was immediately seen, but there was a nonsignificant change in slope toward monthly increase in prescribing of 4% in the postintervention period (IRR, 1.00–1.09; *P =* .06), with a median of 41.12 (IQR, 30.54–46.70) prescriptions per 1,000 PE. During the preintervention period, the median prescribing rate of nitrofurantoin was 28.27 prescriptions per 1,000 PE (IQR, 26.83–30.43), and the postintervention rate was 35.84 prescriptions per 1,000 PE (IQR, 29.93–38.36). No immediate or slope changes were identified by the ITSA. The median prescribing rates for trimethoprim-sulfamethoxazole were 38.98 prescriptions per 1,000 PE (IQR, 32.59–42.45) and 38.59 (IQR, 27.78–42.89) for the pre- and postintervention periods, respectively, without any immediate or slope changes noted in the ITSA.

## Discussion

Our results show that after implementation of cascade reporting of fluoroquinolones, a significant and sustained decrease in prescribing of this antibiotic class was seen at the urgent care and express care clinics affected by the intervention.

Previous studies have shown that cascade reporting in inpatient settings can lead to sustained reductions in the use of the targeted antibiotics^6,7^ and can prompt de-escalation in cases of gram-negative bacteremia.^8^ Regarding outpatient settings, Tan et al^9^ conducted a survey of prescribing practices in England and found that prescribing volumes were higher for antibiotics reported by local microbiology laboratories. In a study by Langford et al^10^ among outpatients aged >65 years in Ontario, Canada, reporting antibiotic susceptibility was associated with increased odds of prescribing the reported antibiotic. We now show that implementation of cascade reporting in outpatient settings can lead to sustained reductions in fluoroquinolone use. Moreover, the Centers for Disease Control and Prevention Core Elements of Outpatient Antibiotic Stewardship identify microbiology laboratories as potential partners for outpatient stewardship activities, and our results suggest that cascade reporting of urine cultures collected in clinics can be implemented as an action for practice to improve antibiotic prescribing.

Our study had several limitations. The study was conducted in a mid-size city with a relatively homogeneous population and low baseline prevalence of antimicrobial resistance. Although the microbiology laboratory is centralized, it is still part of an integrated health system and therefore communication with laboratory personnel is easy. Furthermore, we did not capture clinic or provider-level characteristics nor individual patient-level data to assess diagnostic indication for antibiotics or treatment failure rates associated with specific antibiotic classes. Importantly, from March to September 2020, multiple urgent care and express care clinics had to halt clinical services due to the COVID-19 pandemic. However, the impact on our results is mitigated by using rates in our analysis, as well as seeing the effect of the intervention many months prior to the onset of the pandemic. We also noted that fluoroquinolone use showed a downward trend prior to onset of intervention. Considering that education and cascading were deployed concomitantly, the impact of each individual component of the intervention could not be separated. Lastly, given the quasi-experimental design, we were unable to control for variability in the characteristics of individuals treated at the clinics; however, these factors would not be expected to have significantly changed throughout the study period.

In conclusion, cascade reporting of antimicrobial susceptibility of Enterobacterales in urine cultures is a feasible and sustainable antimicrobial stewardship that can be applied in urgent care and express care clinics in an integrated health system.
